# Psychological stress and strain on employees in dialysis facilities: a cross-sectional study with the Copenhagen Psychosocial Questionnaire

**DOI:** 10.1186/1745-6673-9-4

**Published:** 2014-02-05

**Authors:** Maren Kersten, Agnessa Kozak, Dana Wendeler, Lara Paderow, Matthias Nübling, Albert Nienhaus

**Affiliations:** 1 Institution for Statutory Accident Insurance and Prevention in the Healthcare and Welfare Services, Pappelallee 33-37, 22089 Hamburg, Germany; 2 University Medical Center Hamburg-Eppendorf Competence Centre for Epidemiology and Health Services Research in Nursing (CVcare), Martinistr. 52, 20246 Hamburg, Germany; 3 University of Applied Science in Hamburg, Lohbrügger Kirchstraße 65, 21033 Hamburg, Germany; 4 Freiburg research centre for occupational and social medicine, Bertoldstr. 27, D 79098 Freiburg, Germany

**Keywords:** Belastung, Beanspruchung, Dialyse-Beschäftigte, Gesundheitsberufe, psychosoziale Arbeitsbedingungen, COPSOQ, Stress, Strain, Dialysis staff, Health occupations, Psychosocial factors, COPSOQ

## Abstract

**Background:**

Work in dialysis facilities involves long term contact with chronically ill patients. International comparisons make it clear that dialysis work is being concentrated, staff is being reduced and more patients are being treated. It is more than 20 years since the last German publication on job strains and job satisfaction experienced by dialysis staff was published. The present study examines the stress and strain currently experienced by the staff of German dialysis facilities.

**Methods:**

The staff of 20 dialysis facilities were surveyed with the Copenhagen Psychosocial Questionnaire (COPSOQ). The questionnaire was extended by adding dialysis-specific questions. The data from the dialysis facilities were assessed by comparison with other professions in medical care - nurses and geriatric nurses - using data recorded in the German COPSOQ database.

**Results:**

A total of 367 employees took part in the study, corresponding to a response rate of 55%. For almost all psychosocial aspects, the dialysis staff regarded the stress and strain as being more critical than did the geriatric nurses. There were some positive differences in comparison to hospital nursing, including less conflict between work and private life. However, there were also negative differences, such as fewer possibilities of influencing the work.

**Conclusions:**

The results of the study show that dialysis work exhibits both positive and negative aspects in comparison with other healthcare professions. The results in the different facilities were highly variable, indicating that the deficits found in the individual scales are not inevitable consequences of working in dialysis in general, but are influenced and might be favourably altered by the individual facilities.

## Abstract (de)

**Zusammenfassung:**

**Hintergrund**

Die Arbeit in Dialyse-Einrichtungen ist geprägt durch langfristige Kontakte zu chronisch kranken Patienten. Die Verdichtung der Arbeit in der Dialysetätigkeit, Personalkürzungen sowie die steigende Zahl der Patienten zeigen sich in internationalen Vergleichen. Die letzte deutsche Veröffentlichung zum Thema Arbeitsbeanspruchungen und Arbeitszufriedenheit bei Dialysebeschäftigten ist vor mehr als 20 Jahren publiziert worden. Die vorliegende Studie untersucht die derzeitige Belastungs- und Beanspruchungssituation der Beschäftigten in Dialyse-Einrichtungen in Deutschland.

Eine Befragung von 20 Dialyse-Einrichtungen mit dem Copenhagen Psychosocial Questionnaire (COPSOQ) wurde durchgeführt. Der Fragebogen wurde um sieben dialysespezifische Fragen erweitert. Die gewonnenen Daten aus den Dialyse-Einrichtungen wurden zur besseren Einordnung mit denen anderer Pflegeberufen (Krankenpflege und Altenpflege) verglichen, welche in der COPSOQ-Datenbank erfasst sind.

**Ergebnisse**

Insgesamt nahmen 367 Beschäftigten an der Studie teil (Responserate 55%). Die Dialysebeschäftigten schätzten in fast allen psychosozialen Aspekten die Belastungs- und Beanspruchungssituation kritischer ein als diejenigen in der Altenpflege. Im Vergleich zu der stationären Krankenpflege gibt es zum Teil positive Unterschiede, z.B. geringere Konflikte zwischen Arbeit und Privatleben als auch kritischere Bewertungen, wie z.B. geringere Einflussmöglichkeiten bei der Arbeit.

**Schlussfolgerungen**

Die Ergebnisse der Studie weisen sowohl positive als auch negative Arbeitsaspekte der Dialysetätigkeit gegenüber den anderen Pflegeberufen auf. Die hohe Variabilität der Ergebnisse beim Vergleich zwischen den Einrichtungen deutet darauf hin, dass die beobachteten Defizite in einzelnen Skalen nicht zwingend dialysespezifisch sind, sondern von den konkreten Arbeitsbedingungen in den einzelnen Einrichtungen abhängen und von diesen beeinflusst werden können.

## Background

Work in dialysis facilities is marked by intensive and long-term contact with chronically ill patients [1,2], who are frequently frustrated or depressive [3]. This confrontation with suffering and death is very demanding for the medical care personnel in dialysis facilities [2,4-6]. There are also problems with staff reductions [7,8] and in mastering new modern technology [5,9,10].

In recent years, there have been numerous studies on the psychological stress and strain to which nurses and geriatric nurses are exposed [11-14], but little work has been done on the staff in dialysis facilities [15].

In work science and also in norms for measuring psychological stress, for instance ISO 10075 - one distinguishes between work load and stress (entirety of measurable external influence e.g. social working environment) and strain (effects of the stress on employee depending on his/her individual conditions e.g. emotional exhaustion) as well as the consequences of strain (e.g. disease).

A systematic review on stress and strain in employees in dialysis facilities [16] reported heterogeneous study results on stress, working conditions, strain and burnout. Current scientific knowledge indicates that there are positive aspects of this work, including high job satisfaction [1,7]. Moreover, nurses in dialysis facilities perceived their work as worthwhile and were very interested in professional details [17]. They enjoyed the high responsibility [1], as well as the chance to do things for people, the freedom to use their own judgement and the job security [15]. The negative aspects of dialysis work, as described in the literature, include low involvement in decision making [1,17], pressure at work, lack of time for individual patients, monotonous work and fear of blood-borne diseases [17]. Other negative aspects were the low salary and the general working conditions [15]. “Contact with other staff members could be a stressor as well as a resource for the nurses” [18]. Taken together, the data in the review indicate that the level of burnout is moderate for dialysis employees compared with different groups. In the systematic review seven studies were identified that used the same measurement (Maslach’s Burnout Inventory). Three of these are comparable, because they used the same number of response scales and items. In the study by Klersy et al., burnout is moderate for dialysis employees (nurses and physicians) compared to healthcare workers and the general population in Italy [19]. In a further study, Arikan et al. found moderate burnout levels in comparison with intensive care units and ward nurses [20]. The study of Lewis et al. [5] used the medical workers (nurses and physicians) as reference group. In this study, the burnout level was slightly higher for the dialysis staff than in the reference groups.

The number of patients with chronic renal failure will rise in future, one reason being the increasing number of patients with diabetes. In 2012, about 90,000 persons required dialysis in Germany [21]. It can be expected that dialysis staff will be exposed to increasing stress, as staff numbers are being reduced and the number of patients is increasing, partially due to increased life expectancy [1,8]. It is therefore of the greatest importance that staff in dialysis facilities should be motivated and healthy and stay in their profession for as long as possible.

There are no current analyses of the working situation of dialysis staff in Germany. The last German study on the job strain and job satisfaction of dialysis nurses was published more than 20 years ago [4]. This study yielded the following results: the main sources of stress were the principle of maximum therapy, the consumerism of some patients and intra-team tensions. Almost two thirds of the employee confirmed the necessity of psychosocial staff training.

The objective of the present study is to examine the current psychological stress and strain on dialysis employees. The survey results for the dialysis staff were assessed by comparison with those for hospital nurses and for geriatric nurses to facilitate better interpretation and comparability.

## Methods

### Study sample

The total sample consisted of 20 dialysis facilities. Of these, 14 were from a random sample and six were from an opportunity sample located in North and Central Germany. For the random sample, 30 dialysis facilities insured at the Institution for Statutory Accident Insurance and Prevention in the Healthcare and Welfare Services (BGW) were randomly selected and requested to participate. The survey took place between October 2010 and April 2011. The two samples exhibit a difference in socio-demographics: In the random sample the proportion of women was higher (92.5% vs. 83%, p = 0.009) than in the opportunity sample. The two samples differ only in one scale of the COPSOQ scales, namely in the scale social relations (47 vs. 36, p > 0.001). The study was approved by Hamburg Ethics Committee. Each of the dialysis facilities was sent a report of the results, on the basis of which appropriate measures could be deduced. In addition, the dialysis facilities were offered counselling and support in the context of this study. All facilities accepted these offers.

### Measurement

The survey was performed with the German standard version of the Copenhagen Psychosocial Questionnaire (COPSOQ) - a well validated and internationally recognised survey instrument for the measurement of psychosocial stress at work [22]. The questionnaire includes 87 items, mapped in 25 constructs: 22 scales and three single items. Mostly, a five-point Likert scale is used, where the first category represents the maximal value (for example, “always”) and the last the minimal value (for example, “never”). These categories were allocated point scores (maximum = 100, minimum = 0). This transformation of the categories into point values is in accordance with a standardised procedure that was already used in the German validation study [23].

**Figure 1:**
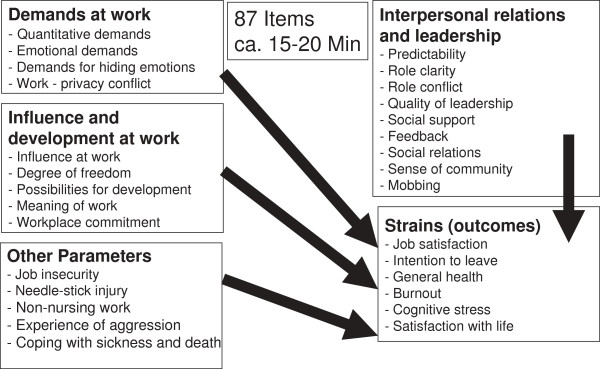
Dimensions of the COPSOQ questionnaire, with some additional dialysis items.

Data from a lot of studies done since 2005 applying this questionnaire is entered into a steadily growing COPSOQ database. In this database the data is weighted und classified by professional groups [24,25]. Surveys with the COPSOQ questionnaire thus allow a comparison of the results with profession specific reference values. The COPSOQ questionnaire consists of four main dimensions: demands at work, influence and development, interpersonal relations and leadership, strain (effects, outcomes) and one additional scale (job insecurity; Figure 1). The professional groups of hospital nurses and geriatric nurses, who were also from Germany, were included for comparison and to facilitate the interpretation of the psychosocial situation at work. At the time of the analysis (01 Oct. 2012), the COPSOQ database included 3,037 hospital nurses and 866 geriatric nurses. Of the group of hospital nurses, 84% were female and 16% male. One third of the employees (33%), were aged 40–49 years. 51% of the hospital employees worked full time. Of the group of geriatric care nurses, 88% of the employees were female and 12% were male. Two fifths of the geriatric care nurses (40%) were aged 40–49 years. 46% of the geriatric care nurses worked full time. In the surveys with the hospital and geriatric care nurses, not all the same information was collected as in the dialysis study.

### Other parameters

To assess dialysis specific working conditions (hazards and demands), this questionnaire was extended by seven questions, which were based on a literature search [16], as well as qualitative interviews with experts. The following three questions had dichotomous response categories (yes/no):

▪ Within the last 12 months, have you had a needle-stick injury?

▪ During the last 12 months, have you experienced physical aggression from a patient?

▪ During the last 12 months, have you experienced verbal aggression from a patient?

Four additional questions could be answered on a five-point Likert scale. The answers ranged from 1 (“to a very high degree”) to 5 (“to a very low degree”):

▪ Are you scared of becoming infected with a blood-borne disease during work?

▪ Do you feel stressed by the verbal or physical aggression of your patients or their families?

▪ Are you expected to perform many tasks that are remote from the patients (e.g. organisation or documentation)?

▪ Are you stressed by having to cope with the patients’ suffering or death?

Persons are rated as highly stressed if they answer the individual dialysis-specific questions with “to a high degree” or “to a very high degree”.

### Statistical analysis

To evaluate the observed differences between dialysis staff, hospital nursing and geriatric nursing, a nomenclature was employed that is already regularly used in COPSOQ studies: a difference of at least 5 points in the mean values of groups is evaluated as a clear difference, a deviation of 10 or more points is evaluated as a very clear deviation [12]. This rule of thumb is based on the effect size measure (Cohen’s d): COPSOQ scales have usually standard deviations of 15–25 points, thus 5 points represent a small to intermediate effect size of 0.2 – 0.33 and 10 point represent middle to strong effect sizes 0.4 to 0.66.

When interpreting COPSOQ scale values it must be borne in mind that high values stand for “a lot” and low values for “a little”. Whether this is favourable or unfavourable depends on the content of the individual scale.

Differences between the professional groups were examined by analysis of variance (ANOVA). Mean value differences were tested with Scheffé’s post hoc test. The values were described as statistically significant if p < 0.05. Only results being statistically significant and fulfilling the 5 points difference criterion (see above) are interpreted. The data analysis was performed with the IBM SPSS Statistics 21 program. The potential influence of different response rates on the study results was assessed by dichotomising the sample in persons from facilities with a response rate below or above the median response rate and performing non- parametric tests for the comparison of distributions of the 25 outcome scales or variables of the COPSOQ. As we have a random and an opportunity sample, the influence of the two different sampling methods was assessed in a similar way.

## Results

A total of 669 questionnaires were issued; 367 employees took part in the study. The response rate was 55%. The interquartile range of the response rate was 45 to 65% and the total range 22 to 96%. With the exception of social relations, no statistically significant difference in the scales and variables of the COPSOQ was observed between the two groups with high or low response rates. The participating dialysis facilities had between 13 and 55 employees. Table 1 describes the study population. Most of the participants were female (90%). The mean age was 43.7 years (±10.4). About one third of the participants was 50 years or older. More than half the interviewees had full time jobs. 27% of the participants performed on-call duties. 40% of the participants stated that they had a night shift at least once a month. Split shifts were rare in the study population (5%). Most participants were registered nurses (71%).

**Table 1:** Description of the sample (N = 367)

**Variable**	**Categories**	**n (%)**
Sex	Female	329 (90%)
	Male	38 (10%)
Age (years)	< 30	57 (16%)
	30–39	64 (17%)
	40–49	125 (34%)
	≥ 50	110 (30%)
	No information	11 (3%)
Type of employment	Full time (> 34 h)	189 (51%)
	Part time (15–34 h)	164 (45%)
	Part time (< 15 h)	13 (4%)
On-call duties (per month)	None	267 (73%)
	1–5 times	89 (24%)
	> 5 times	11 (3%)
Night shifts (per month)	Never	219 (60%)
	1–5 times	118 (32%)
	> 5 times	30 (8%)
Split shifts (per month)	Never	348 (95%)
	1–5 times	12 (3%)
	> 5 times	7 (2%)
Variable shifts (per month)	Never	79 (21%)
	1–5 times	91 (25%)
	> 5 times	197 (54%)
Job experience (years)	≤ 5	96 (26%)
	6–10	77 (21%)
	11–15	55 (15%)
	16–20	66 (18%)
	> 20	73 (20%)
Professional group	Certified nurse	260 (71%)
	Trained nurse	22 (6%)
	Other nurse	25 (7%)
	Other professions (e.g. administration)	60 (16%)

The dialysis-specific questions were answered as follows: 9% reported that they had suffered from needle-stick injuries in the previous 12 months. 11% of employees reported that they were very scared of becoming infected with a blood-borne disease. Though, 26% of employees reported that they were occasionally scared of an infection. 25% of participants reported that they had suffered physical aggression within the previous 12 months. The majority (72%) of employees had suffered verbal aggression. 15% of employees felt under severe stress from physical or verbal aggression from the patients or their families. Almost half the participants (48%) felt that they were under severe stress due to non-nursing tasks. 25% of employees were under stress from coping with the patients’ suffering or death.

**Figure 2:**
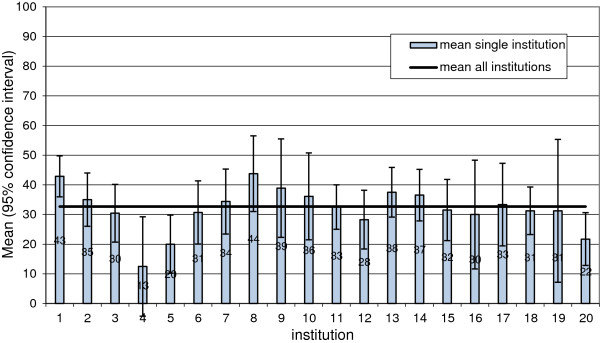
Rating of all dialysis facilities concerning the “fear of blood-borne diseases”.

For the dialysis-specific items, there are no comparative values from other professional groups. Therefore a mean value was calculated for each dialysis facility and compared with the mean for all dialysis facilities. The following illustration shows the variability in the answers among the dialysis facilities, using the question, “Are you scared of becoming infected with a blood-borne disease during work?” as an example. The mean value was 33, with a minimum at 13 points and a maximum at 44 points (Figure 2).

The following table (Table 2) shows the differences in the means for the different COPSOQ scales for dialysis staff, hospital nursing and geriatric nursing.

**Table 2:** Differences in means for the different COPSOQ scales

**Scale**	**COPSOQ dialysis**	**COPSOQ nursing**	**Difference dialysis-nursing**	**p value (Scheffé)**	**COPSOQ geriatric nursing**	**Difference dialysis-geriatric nursing**	**p value (Scheffé)**
	**n = 367**	**n = 3037**			**n = 866**		
	**Mean (SD)**	**Mean (SD)**	**Points**		**Mean (SD)**	**Points**	
Demands at work							
Quantitative demands	55 (16)	60 (17)	−5	< 0.001	51 (21)	4	0.003
Emotional demands	60 (19)	65 (18)	−5	< 0.001	54 (20)	6	< 0.001
Demands for hiding emotions	53 (23)	53 (21)	0	n.s.	43 (22)	10	< 0.001
Work-privacy conflict	48 (25)	53 (27)	−5	0.011	43 (29)	5	0.003
Influence and development at work							
Influence at work	29 (19)	38 (20)	−9	< 0.001	42 (21)	−13	< 0.001
Degree of freedom	33 (16)	40 (19)	−7	< 0.001	41 (19)	−8	< 0.001
Possibilities for development	58 (17)	70 (17)	−12	< 0.001	71 (17)	−13	< 0.001
Meaning of work	77 (17)	80 (17)	−3	0.006	86 (15)	−9	< 0.001
Workplace commitment	55 (18)	55 (19)	0	n.s.	63 (20)	−8	< 0.001
Interpersonal relations and leadership							
Predictability	50 (22)	55 (21)	−5	< 0.001	64 (21)	−14	< 0.001
Role clarity	74 (16)	76 (16)	−2	0.018	80 (15)	−6	< 0.001
Role conflict	44 (20)	48 (21)	−4	0.002	39 (21)	5	0.004
Quality of leadership	47 (24)	54 (25)	−7	< 0.001	64 (24)	−17	< 0.001
Social support	65 (20)	69 (20)	−4	0.002	73 (19)	−8	< 0.001
Feedback	37 (21)	44 (22)	−7	< 0.001	53 (23)	−16	< 0.001
Social relations (quantity)	46 (17)	48 (28)	-2	n.s.	34 (30)	12	< 0.001
Sense of community	73 (16)	76 (17)	−3	0.010	78 (18)	−5	< 0.001
Mobbing	27 (26)	20 (23)	7	< 0.001	19 (23)	8	< 0.001
Insecurity at work	28 (22)	30 (22)	−2	n.s.	31 (24)	−3	n.s.
Strain (effects, outcomes)							
Job satisfaction	62 (14)	61 (15)	1	n.s.	67 (15)	−5	< 0.001
Intension to leave	17 (23)	18 (23)	−1	n.s.	12 (19)	5	0.014
General health	70 (19)	72 (19)	−2	n.s.	70 (20)	0	n.s.
Personal burnout	48 (19)	48 (18)	0	n.s.	42 (19)	6	< 0.001
Cognitive stress	32 (19)	29 (19)	3	n.s.	26 (19)	6	< 0.001
Satisfaction with life	66 (19)	66 (19)	0	n.s.	66 (20)	0	n.s.

*Note.* ≥5 points difference in mean corresponds to a small effect size (0.2-0.33) and ≥10 points to an intermediate or high (0.4-0.66) effect size.

Employees in dialysis can best be compared to hospital employees, as most of the sample had similar education (Table 1). The greatest significant positive effects (dialysis versus hospital nursing) with at least a small effect size were found in the following aspects: lower quantitative demands (55 vs. 60) and lower emotional demands (60 vs. 65), as well as lower work-privacy conflicts (48 vs. 53).

Overall, it was found that the influence and the degree of freedom at work in dialysis facilities was rated markedly lower in dialysis facilities than in hospital nursing: Influence at work (29 vs. 38), degree of freedom at work (33 vs. 40) and possibilities for development (58 vs. 70). As regards the dimension of social relations and leadership, it was found that the quality of leadership (47 vs. 54), feedback (37 vs. 44) and predictability (50 vs. 55) were also rated as being poorer. Dialysis staff also felt that they were more exposed to mobbing (27 vs. 20).

A comparison was also made with geriatric nursing. Staff at dialysis facilities was found to have a more critical view of the stress and strain than the geriatric nurses, with respect to almost all psychosocial aspects. The exception was the quantity of social relations at work, which was viewed much more favourably by the dialysis employees (46 vs. 34).

**Figure 3:**
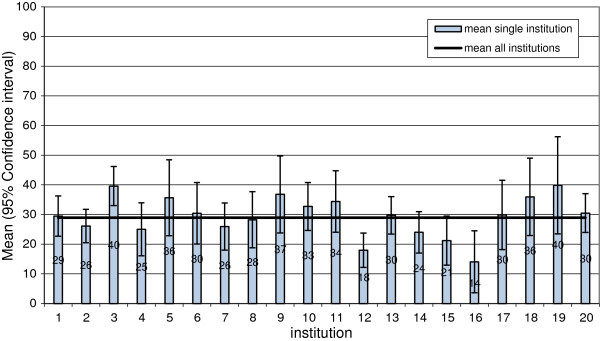
Mean values of 20 dialysis facilities for the scale “influence at work”.

In all the scales, there was striking variability between the individual dialysis facilities (N = 20). For example, the Figure 3 shows the variability between the facilities with respect to the scale “influence at work” (overall mean = 29 points).

## Discussion

This is the first study for more than 20 years to record the psychological stress and strain on dialysis staff in Germany. By means of a survey with the COPSOQ questionnaire, the psychosocial occupational stress and strain were recorded in 367 employees working in 20 different dialysis facilities. To facilitate the classification of the dialysis survey, the results were compared with those for other health care professions. The dialysis results were less favourable than those for institutional geriatric care in almost all aspects. In the comparison with hospital nursing, the dialysis results were more favourable in the dimension of demands at work (quantitative demands, emotional demands and work-privacy conflict). More critical values were mainly found in the dimension influence and development at work (influence at work, degree of freedom, possibilities for development), as well as the dimension interpersonal relations and leadership (predictability, quality of leadership, feedback, mobbing).

The dialysis staff rated the quantitative demands considerably lower than did the geriatric nurses. However, the values for the dialysis staff were the same as for the COPSOQ mean (the weighted mean for all employees who answered the questionnaire; database 01. Oct. 12). In spite of the favourable comparison with hospital nursing, this indicates that the quantitative demands are in need of improvement. Many studies show clearly that quantitative demands are a critical issue, particularly in the context of staff reductions [5,17] or limited number of staff [10,26]. As for the emotional demands, the hospital nurses rated this aspect considerably higher than the other two professional groups. In a study among different professionals groups, jobs with client work scored highest on both aspects: emotional demand and demands for hiding emotions [27]. Ashker et al. examined the work-related emotional stressors among dialysis staff and concluded that four factors influence the emotional well-being: intensive and long-term relationships with the patients, development of the disease and near-death experiences, deterioration in the patient’s physical and psychological well-being and pressure at work [2].

Although some aspects of demands at work were generally rated better in dialysis than in hospital nursing, there is still need for action. The stress may rise in dialysis setting, as a result of possible staff reductions in the health care system, reductions in budgets, the increase in the number of dialysis-dependent patients with multiple diseases, as well as the complex technical handling of modern instruments.

As discussed in Karasek’s demand-control-support model (as extended by: Karasek, R. & Theorell, T. [28]), it is not inevitable that high demands at work are inherently negative, if balanced by high degree of freedom and social support at work. However, the dialysis employees rated their influence and the degree of freedom as being significantly lower than did hospital nurses, thus a compensation of the relatively high demands by high control or support is not given. An obvious conclusion is that the standardized work processes allow little possibility of influencing the work; the employees themselves described their work as routine [17]. Other studies also have shown that dialysis staff would like to be more actively involved in the decision making process [1,17,29]. There are major possibilities for development in this dimension. The view of the 20 dialysis facilities in this survey makes it clear that some facilities exhibit much better values in this respect than do others.

The results indicate that it is easier for dialysis staff to combine work and private life than it is for hospital nurses. Other studies have emphasised night shifts [20] or shift work [18] as stressors. In Germany night shifts are performed by nearly very nurse in hospital and geriatric care. The lower number of dialysis nurses (40%) performing night shifts might explain the positive effect on the compatibility between private and professional lives in our study.

Aspects of the dimension of interpersonal relations and leadership were more critically rated than by hospital nurses (e.g. predictability, quality of leadership, feedback and mobbing). These aspects have hardly been considered or have not been explicitly queried in the literature on the occupational situation in dialysis. Apart from that, these findings provide the first evidence for possible interventions in dialysis facilities. Enhancement of leadership competence and establishment of a constructive feedback culture could lead to improvements in these aspects, supported by better adapted planning and work management, optimised to the requirements of the employees. According to a current research with the COPSOQ dataset, the quality of leadership contributed significantly to job satisfaction [30]. Gregersen et al. found in their review the first empirical evidence on the influence of various leadership style on the health of employees [31]. However, more research is needed on this aspect in the dialysis setting.

As regards the dialysis-specific questions, it was found that 25% of the employees had high or very high fear of blood-borne diseases. In contrast, Brokalaki et al. found that 79% of participants had high fear of blood-borne diseases [1]. Similar results were also found by Nakahara et al. [17]. According to data from the compensation board in Germany (BGW), blood-borne viral diseases have become rarer in health service employees [32]. It is unfortunately unknown how frequently dialysis employees are affected. If safe products are used in dialysis, this may help to alleviate employees’ fear of blood-borne viral diseases.

In our study, the majority of participants (72%) reported that they had experienced verbal aggression during the preceding 12 months. 25% of participants reported that they had suffered physical aggression. In a qualitative study, Murphy reported that most employees had suffered verbal and physical aggression during their working lives [18]. The comparison between dialysis staff and hospital nurses led to the following picture: Schablon et al. recorded that 79% of hospital nurses had suffered verbal aggression [33]. This is consistent with the percentage (72%) in the present study. Moreover, 25% of dialysis staff reported physical aggression, which is much lower than the value found for the hospital nurses (56%). On the other hand, 15% of dialysis staff felt that they were under severe stress due to verbal or physical aggression. It is therefore important that dialysis employees should have training in de-escalation and coping strategies, in particular for situations in which employees have been attacked.

Almost every second dialysis employee felt under severe stress from non-nursing tasks. Lewis et al. also reported occupational stress from non-nursing tasks [5]. Other studies do not differentiate between nursing and non-nursing tasks, but just report high occupational stress [1,8,17].

Twenty-five per cent of participants were stressed by having to cope with sickness and death. Other studies have found higher values for stress from near-death experiences or the death of patients [1,2,5,6].

When comparing dialysis staff with geriatric nurses, it should be considered that in the COPSOQ database the professions or occupations are classified according to the system of job classification of the German Federal Statistical Office. Thus, there is no differentiation between out-patient and in-patient geriatric care. The values for both sectors are combined to a single mean value, which can bias our conclusion. In a study by Nübling et al. these two groups were separately examined [12]. They found that out-patient personnel had a much better opinion of their psychosocial situation than did in-patient personnel. However, we were surprised by the relatively poor results for dialysis staff in comparison to geriatric nurses in nearly all aspects. It would be interesting to analyse whether and to what extent these differences can be explained by individual *characteristics* or by differences in the organisations.

To our knowledge, there is no comprehensive data to compare our sample with the total population of German dialyses employees. Therefore, we cannot proof the representativeness of the study sample. In the present cross-sectional study, stress and strain were recorded at the same time, which can lead to a common method bias. Moreover, mean comparisons cannot be used to identify factors that may be linked to possible impairments in well-being, i.e. no relationships are established between psychosocial occupational stress and outcomes. Nevertheless, the results provide some indications of possible approaches to reduce stress on employees in dialysis.

## Conclusions

In conclusion, dialysis staff rated their possibilities for influence and development, the social relations and quality of leadership poorer than did hospital and geriatric nursing. Improvement in feedback culture and emphasis by superior on greater employee participation and involvement could decrease the stress suffered by dialysis employees, as the differences from hospital and geriatric nursing are greatest in these areas. The high variance of the results between the facilities indicates that the observed deficits are not inherent to dialysis, but can be favourably influenced by the individual facilities.

## Abbreviations

BGW: Institution for Statutory Accident Insurance and Prevention in the Healthcare and Welfare Services in Germany (Berufsgenossenschaft für Gesundheitsdienst und Wohlfahrtspflege); COPSOQ: Copenhagen Psychosocial Questionnaire.

## Competing interests

The authors declare that they have no competing interests.

## Authors’ contributions

AN and MK conceived the study after a systematic review. Data collection and analysis were performed by MN, AK, DW and MK. The first draft of the paper was written by: MK and AK. Important suggestions for the improvement of the first draft were provided by DW, LP, MN and AN. All authors read and approved the manuscript.
